# A New Approach for Glyco-Functionalization of Collagen-Based Biomaterials

**DOI:** 10.3390/ijms20071747

**Published:** 2019-04-09

**Authors:** Ines Figuereido, Alice Paiotta, Roberta Dal Magro, Francesca Tinelli, Roberta Corti, Francesca Re, Valeria Cassina, Enrico Caneva, Francesco Nicotra, Laura Russo

**Affiliations:** 1 Department of Biotechnology and Biosciences, University of Milano-Bicocca, 20126 Milan, Italy; ines.figueiredo21@hotmail.com (I.F.); alice.paiotta@unimib.it (A.P.); francesco.nicotra@unimib.it (F.N.); 2 School of Medicine and Surgery, University of Milano-Bicocca, 20854 Vedano al Lambro, Italy; roberta.dalmagro@unimib.it (R.D.M.); francesca.tinelli@unimib.it (F.T.); r.corti9@campus.unimib.it (R.C.); francesca.re1@unimib.it (F.R.); valeria.cassina@unimib.it (V.C.); 3 Department of Material Science, University of Milano-Bicocca, 20126 Milan, Italy; 4 UNITECH COSPECT: Comprehensive Substances characterization via advanced sPECTtrometry, 20133 Milan, Italy; enrico.caneva@unimi.it

**Keywords:** biofunctionalization, glyco-microenvironment, cell fate, ECM mimetics, tissue engineering

## Abstract

The cell microenvironment plays a pivotal role in mediating cell adhesion, survival, and proliferation in physiological and pathological states. The relevance of extracellular matrix (ECM) proteins in cell fate control is an important issue to take into consideration for both tissue engineering and cell biology studies. The glycosylation of ECM proteins remains, however, largely unexplored. In order to investigate the physio-pathological effects of differential ECM glycosylation, the design of affordable chemoselective methods for ECM components glycosylation is desirable. We will describe a new chemoselective glycosylation approach exploitable in aqueous media and on non-protected substrates, allowing rapid access to glyco-functionalized biomaterials.

## 1. Introduction

Tissue engineering relies on the possibility to engineer cell microenvironments by means of bioactive materials providing biochemical and physical stimuli to guide cell behavior and to regenerate damaged tissue [1,2,3]. Synthetic scaffolds mimicking different cell microenvironments are therefore important tools to guide cell developments in a tailored mode, inducing specific tissue regeneration. Bioactive materials can be designed and also formulated by the incorporation of specific biological signals, using both peptides or glycans able to control cell adhesion or differentiation. In particular, glycans represent an interesting class of biomolecules due to their ubiquity in tissues, and more importantly, due to their functional and structural roles in cell development. Glycans are strongly involved in physiological and pathological processes, acting as mediators during cell-cell and cell-ECM interactions [4]. 

Several interesting observations have been made in this context, functionalizing with glycans natural and synthetic bio- and nanomaterials [5]. Glycosylated collagen 2D films, obtained via reductive amination of a glycan with the collagen lysins, showed that glycosylation of the biomaterials influences the cell fate [6]. In more detail, collagen 2D films neoglycosylated with glucose are able to drive F11 neuroblastoma cell line from proliferation to differentiation into functionally active neurons [7]. On the contrary, 2D films glycosylated with sialic acid residues induce major proliferation. Interestingly, minor changes in the glycan structure, such as the differences between 3’-sialyllactose versus 6’-sialyllactose, dramatically modifies the cell behavior. We observed that multipotent mesenchymal stem cells (mMSCs) cultured on 2D films exposing 3’-sialyllactose induce expression of osteogenic genes, whereas 2D films exposing 6’-sialyllactose induce expression of chondrogenic genes. It is now clear that the natural glyco-microenvironment can provide inspiration to design new bioactive materials for regenerative medicine, and more in general for cell cultures. Due to the heterogeneity and complexity of glycans, new efficient and chemoselective strategies are needed to glyco-functionalize biomaterials in aqueous media, avoiding protection, activation, and deprotection steps. 

**Scheme 1:**
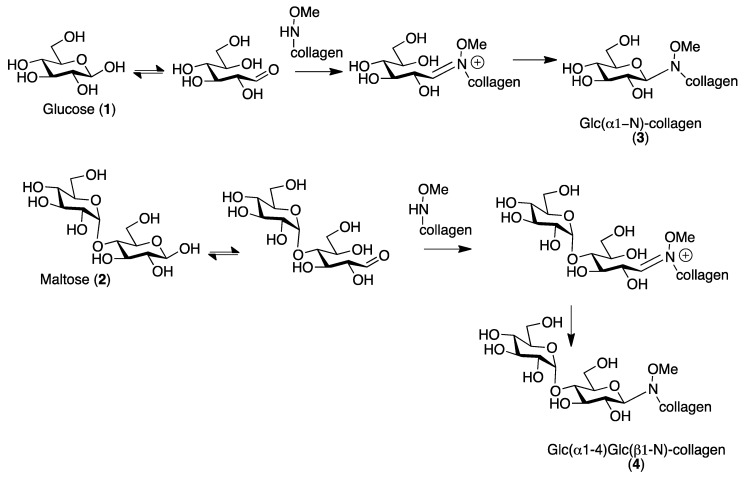
*N*-methoxamino- chemoselective protocol.

Here in this work, we exploited on collagen our *N*-methoxamino- chemoselective protocol [8] that allows for the one-pot conjugation of any reducing glycan. As proof of concept, the reaction has been performed with glucose and maltose. The reaction of glucose (**1**) with N-methoxyamino-collagen provides the β-*N*-glucopyranoside (**3**) due to the anti-anomeric effect that governs the reaction (Scheme 1), and therefore a β-Glc is exposed. Using maltose (**2**), if the reaction generates a β-*N*-glycosydic linkage, the exposed sugar is the terminal α-Glc (**4**).

The biocompatibility of neoglycosylated films has been tested on human SYSH-SY5Y neuroblastoma cell lines.

## 2. Results

### 2.1. Collagen Matrix Neoglycosylation

Neoglycosylation of collagen has been performed using collagen films produced by a solving casting method as previously described [7,9]. 

**Figure 1:**
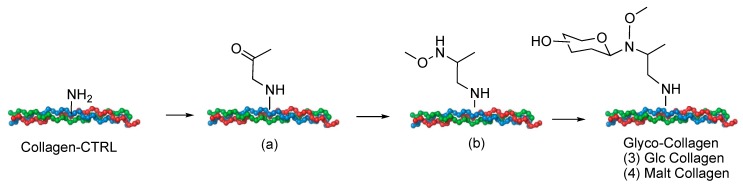
The general scheme of collagen neoglycosylation.

As proof of concept, glucose, usually present on collagen as natural *O*-glycosylation, has been selected. The neoglycosylation protocol will generate a β-glucosidic linkage with N-methoxyamino-collagen. Considering that the exposed glucosidic residue with α or β anomeric configuration, can induce different cell fates, maltose (Glcα1-4Glc), has been also used in order to generate collagen exposing an α-Glc residue (Figure 1). 

The glycosylated materials have been characterized in terms of chemical and morphological features. The biocompatibility of glycosylated films has been tested on human SYSH-SY5Y neuroblastoma cell lines and compared with traditional reductive amination strategies already reported in the literature [7]. 

The quantitative estimation of the neoglycosylation reaction was determined using a sulphuric acid–phenol assay [10,11,12]. The assay has been extensively used in the literature for the quantitative analysis of carbohydrates in complex films and tissues. It entails the hydrolysis of carbohydrates via concentrated sulfuric acid to produce furfural derivatives detectable as a yellow complex by reaction with phenol. Hence, the concentration of carbohydrates can reliably be quantified by extrapolating corresponding values from constructed calibration curves. Exploiting this protocol, we quantified the functionalization of 2 × 1 cm collagen sheets in 67 μg/cm^2^ for glucose (Sample 3 Glc) and 56 μg/cm^2^ for maltose (Sample 4 Mal).

### 2.2. Biomolecular and Morphological Characterization

**Figure 2:**
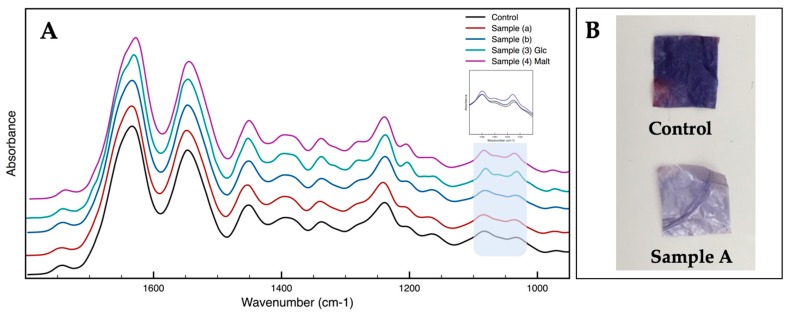
(**A**) FT-IR spectra of collagen samples; (**B**) Ninhydrin assay.

Fourier transform infrared (FTIR) spectroscopy was used to compare the different reaction steps (Figure 2A). The absorbance spectra are comparable for all the samples analyzed. Collagen samples show characteristic FT-IR spectra, with absorption bands of amide I at ∼1650 cm^−1^ and amide II at ∼1560 cm^−1^ [13]. In Samples 3 and 4, a slight increase in the ranges of carbohydrate moiety between 1000–1200 cm^−1^ is noticeable, in particular the typical peak of glucose at 1040 cm^−1^ [13]. Ninhydrin assay has been also performed in the first step to have a fast qualitative method to characterize the success in the insertion of keton groups on the collagen surface (Figure 2B) [14].

**Figure 3:**
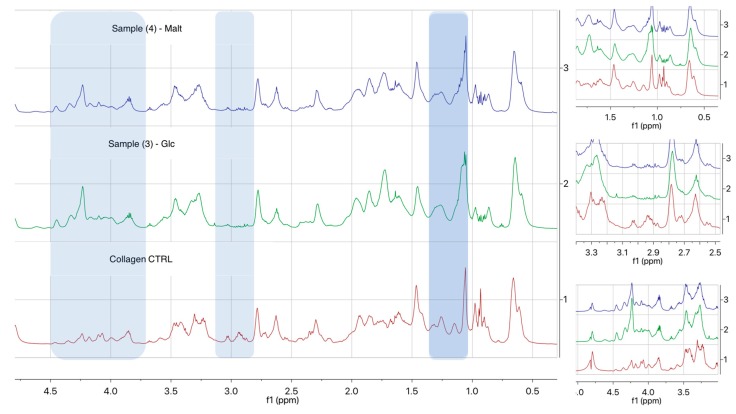
H^1^ NMR spectra of collagen samples.

1H-NMR spectroscopy (Figure 3) displayed the presence of peaks between 2.9 and 3.0 ppm, assigned to the methylene hydrogen of lysine amines that disappear once obtained collagen neoglycosylation for Samples 3 and 4 and the introduction of new signals: One between 1.0–1.2 ppm, attributable to the CH_3_-CHNH of the linker, and a second one around 4.2–4.3 ppm, attributable to the CH_3_-O-NH of the linker, downfield shifted probably for spatial interactions with collagen functional groups. It is also worth noting that the small number of glycans available for the analysis and the heterogeneous nature of the samples presented difficulties regarding sensitivity by NMR. However, the presence of glycans on Samples 3 and 4 can be also be observed in the additional increment of the signals in the spectra between 4.0 and 4.5 ppm, in particular at 4.3–4.4 ppm.

**Figure 4:**
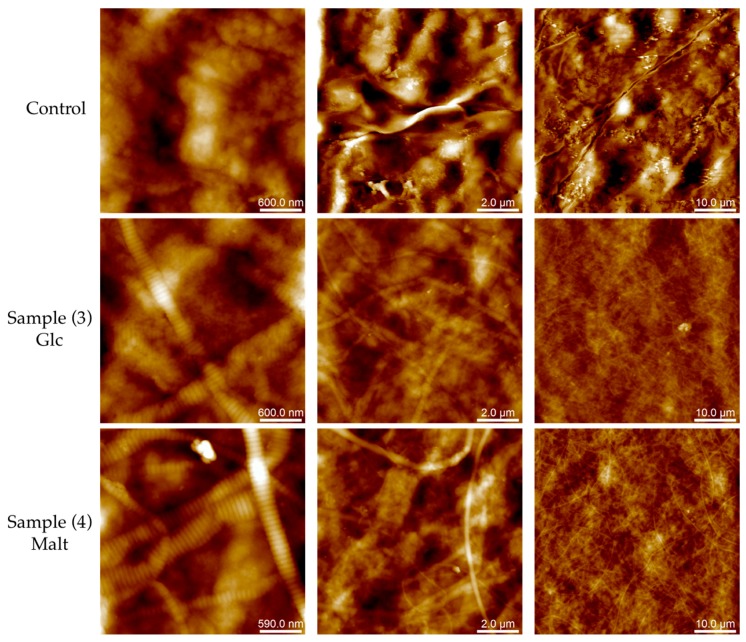
Representative atomic force microscopy (AFM) topography image of collagen films acquired in PeakForce mode in air. Different scan sized images of collagen matrix without further modification, glucose (line 2) and maltose (line 3) neoglycosylated collagen films were collected. From **left** to **right**: column 1 (3 × 3 µm^2^, 512 × 512 pixel, Z-scale 200 nm), column 2 (10 × 10 µm^2^, 512 × 512 pixel, Z-scale 500 nm), and column 3 (50 × 50 µm^2^, 512 × 512 pixel, Z-scale 1.4 µm). For 3D Lighting images and different Z-scale range see Figures S3 and S4 respectively.

Regarding the structural assembly of collagen films, analyzed by atomic force microscopy (AFM), both neoglycosylated collagen samples and pristine collagen reveal significant fibrillation, while the pristine collagen (CT) showed an amorphous surface. As highlighted in Figure 4, Sample 3 and Sample 4 present well-defined fibrils, having respectively pitches of 68 ± 5 nm (Sample 3) and 72 ± 2 nm (Sample 4), regardless of the width of the fibril (see Figures S1 and S2). 

Despite the presence of the fibrils, the roughness of the CT sample (Rq = 196± 44 nm) is approximately two times bigger than the neoglycosylated collagen films (Rq = 74 ± 15 nm for Sample 3 and Rq = 111 ± 25 nm for Sample 4) (see Supplementary Materials, Section 2, for details).

### 2.3. The Effect of Collagen on Cell Viability and Differentiation

**Scheme 2:**
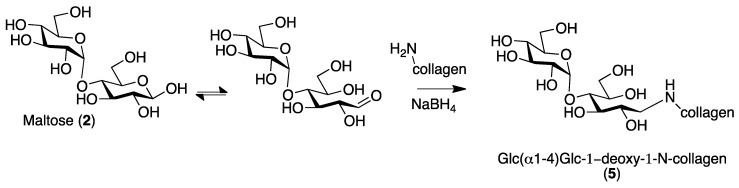
Reductive amination reaction for neoglycosylation of collagen.

Both neoglycosylated Samples (3) Glc-collagen and (4) Malt-collagen were evaluated for their biocompatibility and compared with control and collagen functionalized with α-Glc by reductive amination exploiting maltose (Sample 5) (Scheme 2) [7,15]. 

**Figure 5:**
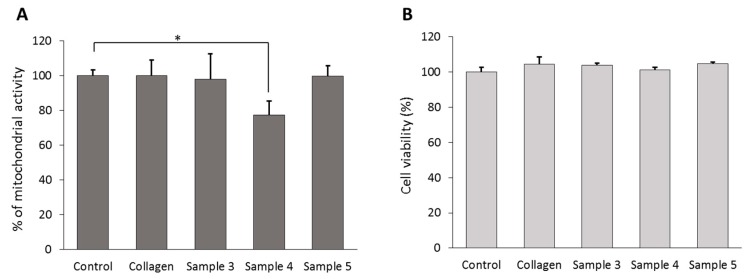
Viability of SHSY5Y cells after 48 h of growth on collagen films. (**A**) MTT assay data and (**B**) LDH assay results for human neuroblastoma cells cultured in normal growth conditions (plastic support) or on different collagen films. Error bars represent SEM. * *p* < 0.05 by Student’s *t*-test.

The viability of cells grown for 48 h on collagen films was evaluated by MTT and LDH assays. Results showed that the mitochondrial integrity and the activity of SHSY5Y cells were not affected by the presence of collagen and glycosylated Samples 3 and 5, while a 23 ± 5% reduction of cell metabolism was observed for cells grown on Sample 4 (Figure 5A). LDH assay results showed that none of the tested collagen films induced a decrease of cell viability, compared to normal growth conditions (Figure 5B).

### 2.4. Evaluation of Cell-Collagen Adhesion

**Figure 6:**
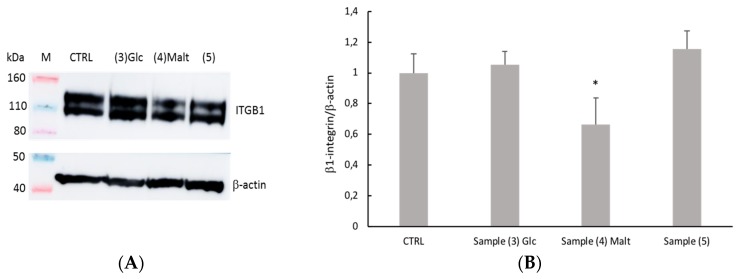
Expression of ITGB1 in differentiated SHSY5Y cells after growing on different collagen films. SHSY5Y cells were cultured on collagen samples for 7 days and subjected to total protein extraction. The total protein extract was analyzed by SDS-PAGE/WB and probed by anti-ITGB1. (**A**) WB of the total cell protein extracts analyzed for b-actin and ITGB. (**B**) The intensity of chemiluminescent bands was semi-quantitatively estimated with Amersham Imager 600 and expressed as the ratio between the intensity of the spot of interest (ITGB) and the intensity of β-actin bands. Data are mean ± SD and compared by Student’s t-test (* *p* < 0.05).

The cell-collagen adhesion has been evaluated by measuring the level of Integrin-β-1 (ITGB), which is the most important cell surface collagen receptor involved in cell adhesion, using Western Blot analysis. The level of expression of ITGB1 decreases only when cells were seeded on Sample 4 (Figure 6). This result could be related to a decrease in viability for the same sample.

## 3. Discussion

Biomaterial neoglycosylation is a new and affordable way to obtain major insight into the role of glycans in cell development under physiological and pathological conditions. Despite the presence of several reports on tools able to recapitulate cell-cell interactions mediated by glycans, their role in ECM–cell and ECM–ECM interactions is less investigated due to the lack of affordable models. 

Collagen is an optimal biomaterial to test alternative methods for the following reasons: it is one of the most used structural proteins in tissue engineering strategies due its ubiquitous presence in the ECM of different tissues and its structural properties, and it can be formulated with different materials to obtain smart hybrid materials. Important results have been already obtained using collagen or other biomaterials neoglycosylated by traditional reductive amination [6]. In this procedure, however, the cyclic structure of the sugar linked to the biomaterial is lost, limiting the applicability of the method. The proposed strategy allows for chemoselective conjugation to a *N*-methoxyamino-functionalised collagen of an unprotected glycan in aqueous media, maintaining its cyclic structure. Exploiting these protocols, collagen sheets have been functionalized with β-Glc (Sample 3) (67 μg/cm^2^) and with α-Glc(1,4)β-Glc (Sample 4) (56 μg/cm^2^). 

AFM analysis reveals that both neoglycosylated films (Samples 3 and 4) have well-defined fibrillary structures, and on the contrary, the untreated control shows amorphous structures. The fibrillary organization of collagen is a fundamental feature in cell culture and tissue engineering. Collagen molecules are packed in a quarter-staggered fashion which gives rise to a repeating banding pattern, the so-called D-periodicity or D-band, of about 67 nm [16,17]. It has been reported in the literature that the transverse D-banding periodic pattern is a key player with respect to fibril mechanical properties, which significantly impacts cell-collagen interactions and is correlated with pathological conditions. In nature, the structural, mechanical, and functional features of native collagen in the ECM are strongly linked to the contribution of several factors. These include a) the interactions with other ECM proteins and soluble proteins (interactors), b) the interaction with cell surface receptors, and c) post-translational modifications (i.e, in collagen type I, the *O*-glycosylation at the hydroxylysine residues). Data reported in the literature on collagen triple helix formation [18] suggests that the glycosylation of collagen chains has an important contribution to surface roughness variations in cell–ECM and ECM–ECM interaction [19,20]. As verified by AFM analysis, morphological changes in neoglycosylated collagen could have an influence on both the inter-molecular and inter-fibrillar interactions of the triple-helical domain of collagen films, contributing also to the improved biological activity of the produced films.

In order to study the biocompatibility of neoglycosylated collagen films, preliminary biocompatibility assay was performed on a human SH-SY5Y neuroblastoma cell line. All the tested neoglycosilated films showed comparable biocompatibility, even though SH-SY5Y cells grown on Sample 4—Malt—showed reduced mitochondrial activity compared to the other samples. The observed alteration of cell metabolic activity could be related to the decrease of ITGB1 expression.

## 4. Materials and Methods 

### 4.1. General Materials

Anhydrous solvents were purchased from Sigma-Aldrich and stored under argon over molecular sieves. Reagents were purchased from standard suppliers and used without further purification. Milli-Q water mQ was obtained with 18.2 MΩ cm at a 25 °C purity.

### 4.2. Collagen Samples Preparation

#### General Procedures

Type I collagen from Bovine Achilles tendon was purchased from Sigma Aldrich. Collagen films were produced using a solvent casting method, as previously described in Reference [9]. At the end of each reaction step, all the samples were washed with 70 mL H_2_O (three times × 15 min) and 30 mL of EtOH (one time × 15 min). After the washes, all the samples were dried under a hood at room temperature for 30 min. 

Sample (a): Collagen matrix (90 mg, 8 × 12 cm) was covered by 20 mL of PBS pH 7.4 and put under gentle agitation. Chloroaceton (290 μL) was added and stirred overnight. The sample was washed and dried and used for the subsequent reaction. 

Sample (b): Sample (a) has been covered by 20 mL of PBS pH 8, and methoxylamine hydrochloride (284 mg) was added and the reaction was left under agitation overnight. After the wash, NaBH_3_CN (207 mg) was added in 20 mL of citrate buffer of pH 6.00 and the reaction was stirred for 24 h. The obtained Sample was washed and dried.

Samples (3) and (4). Neoglycosylation reactions were performed covering Sample (b) with 20 mL of Acetate Buffer at pH 4.00, Glucose (617 mg), and Maltose (1.172 g). The reaction was stirred for 24 h and the obtained sample was washed and dried.

### 4.3. Ninhydrin Assay

A solution of ninhydrin (2 mL, 10 mg/mL) in ethanol was added to both the control collagen and Sample (a). The samples, immersed in ninhydrin solution, were incubated for 5 min at 90 °C, and after cooling to room temperature, were removed from the solution and washed with ethanol. 

### 4.4. Phenol–Sulfuric Acid Assay

To Samples (3) and (4) (2 cm × 1 cm) was added 600 μL of concentrated sulfuric acid. After mixing 30 min, 120 μL of 5% phenol in water was added to the samples. After incubating for 5 min at 90 °C in a static water bath, the samples were cooled to room temperature for 5 min to measure absorbance at 490 nm.

### 4.5. FT-IR

All the FT-IR spectra were recorded in attenuated total reflection ATR mode on a Varian 660-IR instrument. All the samples have been coated on a steel surface and analyzed on different points of the film. The absorbance of the samples and background were measured using 64 scans each. The absorption spectral range was collected between 1800 cm^−1^ and 650 cm^−1^ at a spectral resolution of 2 cm^−1^.

### 4.6. ^1^H NMR

All the NMR ^1^H experiments were carried out on a Bruker Avance I 600 spectrometer (1H base frequency = 600 MHz) equipped with a superconducting Ultrashield Plus magnet of 14.1 Tesla, using a 5-mm BBI reverse broadband probe.

1H spectra were acquired, not spinning, with the presaturation of the residual H_2_O signal (zgpr Bruker sequence), using the following parameters: Spectral width (sw) = 6000 Hz, acquisition time (at) = 2.72 s, number of data points in t2 (TD) = 32k, relaxation delay (d1) = 5 s, number of scans (ns) = 200 and T = 303 K.

### 4.7. Atomic Force Microscopy (AFM) 

AFM measurements were performed in air using a Multimode 8 AFM (Bruker Corporation, Santa Barbara, CA, USA). Images were acquired in peak force tapping mode (PeakForce-Quantitative Nano-Mechanics, PF-QNM). Collagen films were immobilized on a glass surface by letting evaporate a drop of water. V-shaped Scan Asyst Fluid + (0.7 N/m) cantilevers were used. Data processing was performed using the commercial Nanoscope Analysis software (Bruker Corporation, Santa Barbara, CA, USA).

### 4.8. Biocompatibility of Collagen Films 

SH-SY5Y human neuroblastoma cells were cultured in DMEM (Gibco, ThermoFisher Scientific, Milan, Italy) supplemented with 10% FBS (Euroclone, S.p.A., Milan, Italy), 2 mM *L*-glutamine (ThermoFisher Scientific), and 1% penicillin-streptomycin (ThermoFisher Scientific). Cells were maintained at 37 °C, 5% CO_2_, and at saturated humidity. To test collagen biocompatibility with SH-SY5Y cells, a film of collagen, sample 3, 4, or 5 was placed in each bottom of a 24-well plate, washed in 70% ethanol and sterilized by UV irradiation. SHSY5Y cells were grown for 48 h on collagen films (5 × 10^4^ cells/well) and then MTT and LDH assays were performed according to the manufacturer’s protocols. Cells grown on plastic (in absence of collagen film) were used as controls. Optical density values were measured using a SPECTROstar Nano spectrometer (BMG LABTECH, Euroclone Spa, Milan, Italy). Three replicates were used for each condition. 

### 4.9. Immunofluorescence Labeling

SH-SY5Y cells were seeded in 12-well plates (1 × 10^5^ cells/well), previously coated with the different type of collagen films, and cultured for 9 days in complete medium. Then, cells on collagen films were fixed for 20 min with 10% formalin solution, incubated for 25 min with gelatin dilution buffer 1× (GDB; 0.4% (*w*/*v*) gelatin, 40 mM sodium phosphate buffer, pH 7.2, 0.9 M NaCl, 0.2% (*v*/*v*) Triton X-100) to perform permeabilization and blocking and incubated with mouse anti β-III tubulin antibody (1:250; Promega Italia Srl, Milan, Italy) in GBD 1× for 2 h at room temperature. After washes, cells were incubated with Texas Red-conjugated goat anti-mouse IgG (1:200; ThermoFisher Scientific) in GDB 1× for 1 h at room temperature. Four μM Hoechst 33342 (ThermoFisher Scientific) was used to stain nuclei. Finally, the collagen films were mounted on glass slides using ProLong Gold Antifade Reagent (ThermoFisher Scientific). Images were acquired using an inverted confocal laser scanning microscope equipped with a Plan-Neofluar 63 × 1.4 oil objective (Carl Zeis Meditec AG, Jena, Germany). Excitation was performed using an Ar-laser diode (540 nm) and an ultraviolet 25 mV laser diode (405 nm). The pinhole was set to 1 AU.

### 4.10. Integrin-β-1 Immunostaining

SH-SY5Y cells were seeded in 6-well plates (130.000 cells/well), previously coated with the different types of collagen films, and cultured for 7 days in complete medium. Cells were detached with a scraper, then gently transfer into a microcentrifuge tube. Cell suspension was centrifuged at 2.000 rpm for 5 min at 4 °C. Then, RIPA buffer, added with proteases and phosphatase inhibitors, was added to the pellet for 30 min in order to obtain the total protein extract. Samples were centrifuged at 13.000 rpm for 5 min at 4 °C, and the supernatant was analyzed by SDS-PAGE/WB. ITGB1 was identified by immunostaining using anti-ITGB1 antibody (1:1000) and HRP-conjugate secondary antibody. Bands were visualized by chemiluminescence. Band intensities were optically semi-quantified and normalized over beta-actin, as described. 

## 5. Conclusions

In summary, we presented the application of N-methoxamino- chemoselective protocol on collagen films. Maltose and glucose were conjugates to N-methoxyamino-collagen films and the final neoglycosylated materials were characterized in terms of chemical and morphological features. In particular, AFM analysis on neoglycosylated films showed that both the neoglycosylated samples present well-defined fibrils, whereas the control shows an amorphous structure. Preliminary biological assays have been performed on human SH-SY5Y neuroblastoma cell line, to define the biocompatibility of the neoglycosylated collagen films. Also if all the tested samples showed a comparable biocompatibility, the sample (4) functionalized with maltose showed a reduced mitochondrial activity and a decrease of ITGB1 expression that could be related to different recognition phenomena. To clarified how glycosylation of ECM components influences cell fate, further studies on neoglycosylated are needed.

## Supplementary Materials

Supplementary materials can be found at https://www.mdpi.com/1422-0067/20/7/1747/s1.

## Abbreviations

NMR	Nuclear Magnetic Resonance
DOSY	Diffusion Ordered SpectroscopY
FT-IR	Fourier Fourier Transform Infrared Spectroscopy
AFM	Atomic Force Microscopy
Malt	Maltose
Glc	Glucose
