# Supplementary material for: A New Approach for Glyco-Functionalization of Collagen-Based Biomaterials
Source: Int J Mol Sci. 2019 Apr 9;20(7):1747. doi: 10.3390/ijms20071747 (PMC6480084; doi:10.3390/ijms20071747)
Supplement: Supplementary file 1 [file ijms-20-01747-s001.pdf]

SUPPLEMENTARY MATERIAL

A New Approach for Glyco-Functionalization of Collagen-Based Biomaterials

Ines Figuereido [1], Alice Paiotta [1], Roberta Dal Magro [2], Francesca Tinelli [2], Roberta Corti [2,3], Francesca Re [2], Valeria Cassina [2], Enrico Caneva [4], Francesco Nicotra [1] and Laura Russo [1,*]

1. AFM analysis

a)

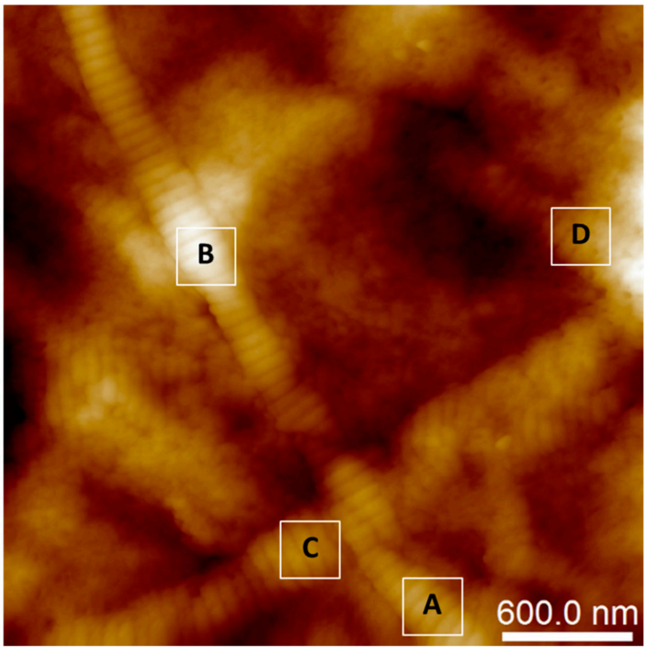

b)

	Pitch (nm)	Width (nm)
Sample 4 A	70±3	237
Sample 4 B	65±4	269
Sample 4 C	75±5	388
Sample 4 D	61±4	293

c)

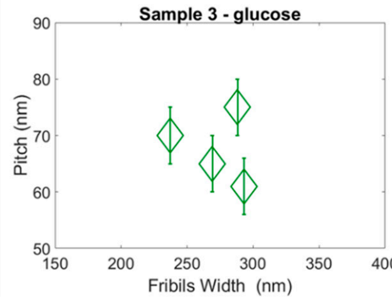

Figure Supplementary 1: a) 3x3 µm2 AFM topography image of Sample 3 (Glc) reported in the main text (Figure 4) with the indication of the chosen fibrils for the pitch analysis, **b)** the pitch and the width of each fibrils and **c)** the absence of correlation of the pitch with the fibril's width

a)

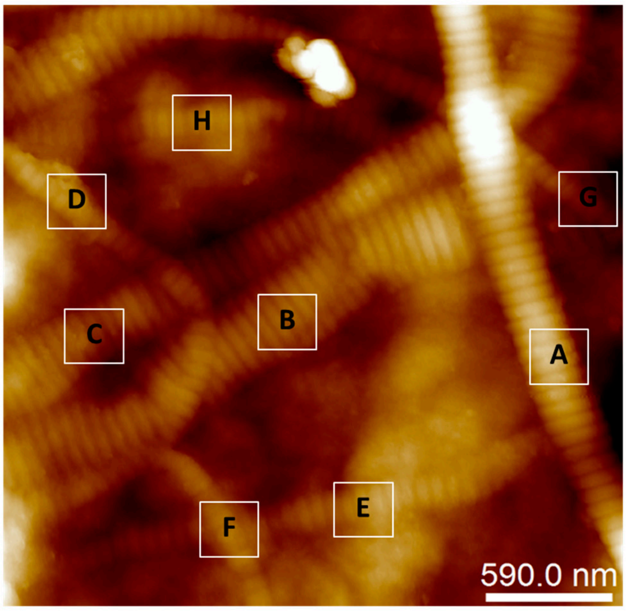

b)

	Pitch (nm)	Width (nm)
Sample 4 A	73±1	307
Sample 4 B	71±1	371
Sample 4 C	70±1	346
Sample 4 D	73±3	193
Sample 4 E	73±3	216
Sample 4 F	73±3	178
Sample 4 G	73±3	165
Sample 4 H	73±3	178

c)

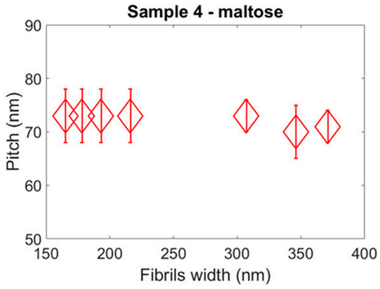

Figure Supplementary 2. a) 3x3 μm2 AFM topography image of Sample 4 (Malt) reported in the main text (Figure 4) with the indication of the chosen fibrils for the pitch analysis, **b)** the pitch and the width of each fibrils and **c)** the absence of correlation of the pitch with the fibril's width.

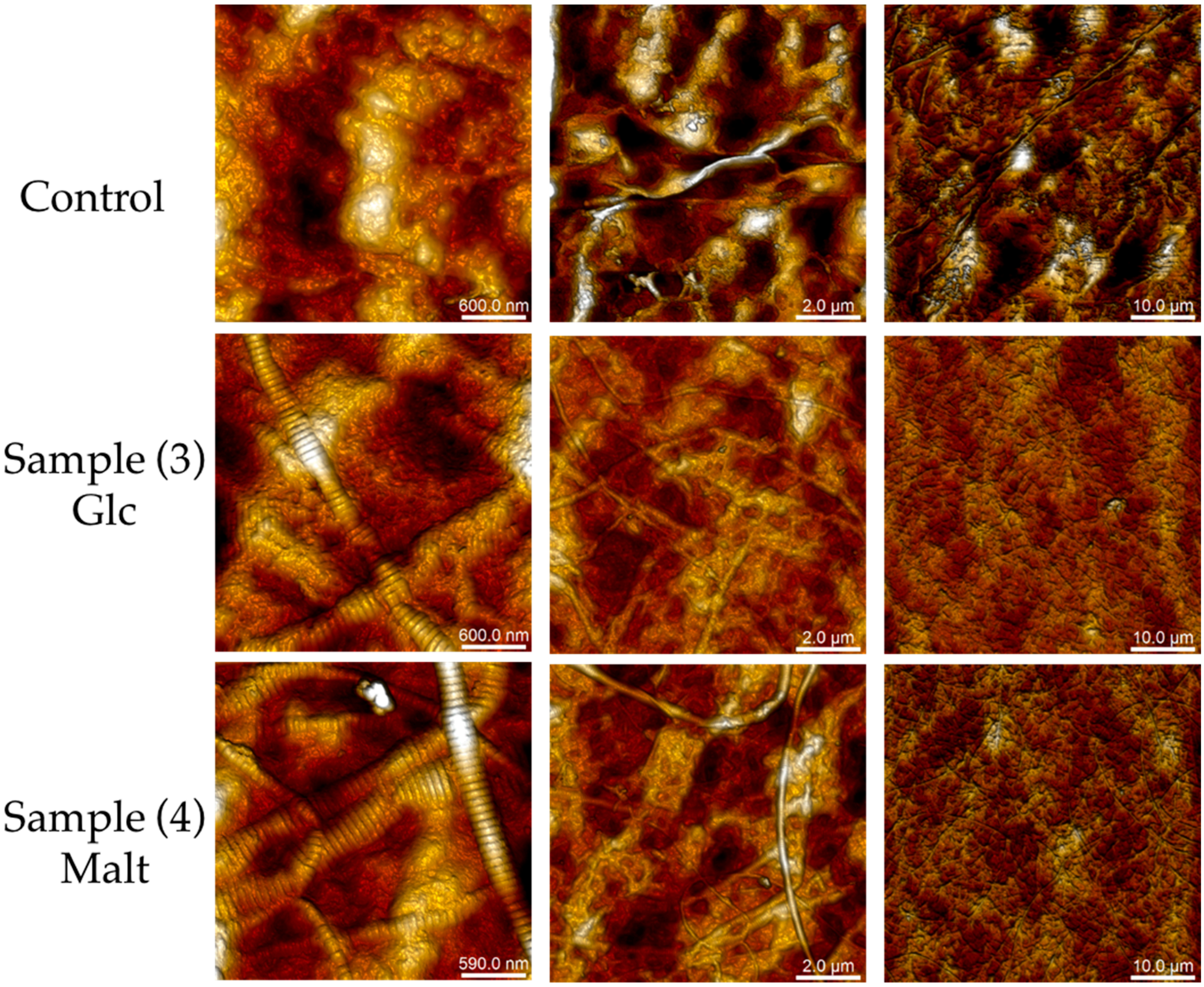

Figure Supplementary 3. 3D Lighting (Nanoscope Analysis software -Bruker Corporation, Santa Barbara, CA) image of collagen matrices acquired in PeakForce mode in air reported in the Figure 4. Different scan sized images of collagen films without further modification (line 1), glucose (line 2) and maltose (line 3) neoglycosylated collagen films were collected. From left to right: column 1 (3x3 µm2, 512 x 512 pixel, Z-scale 200nm), column 2 (10x10 µm2, 512 x 512 pixel, Z-scale 500nm), column 3 (50x50 µm2, 512 x 512 pixel, Z-scale 1.4 µm).

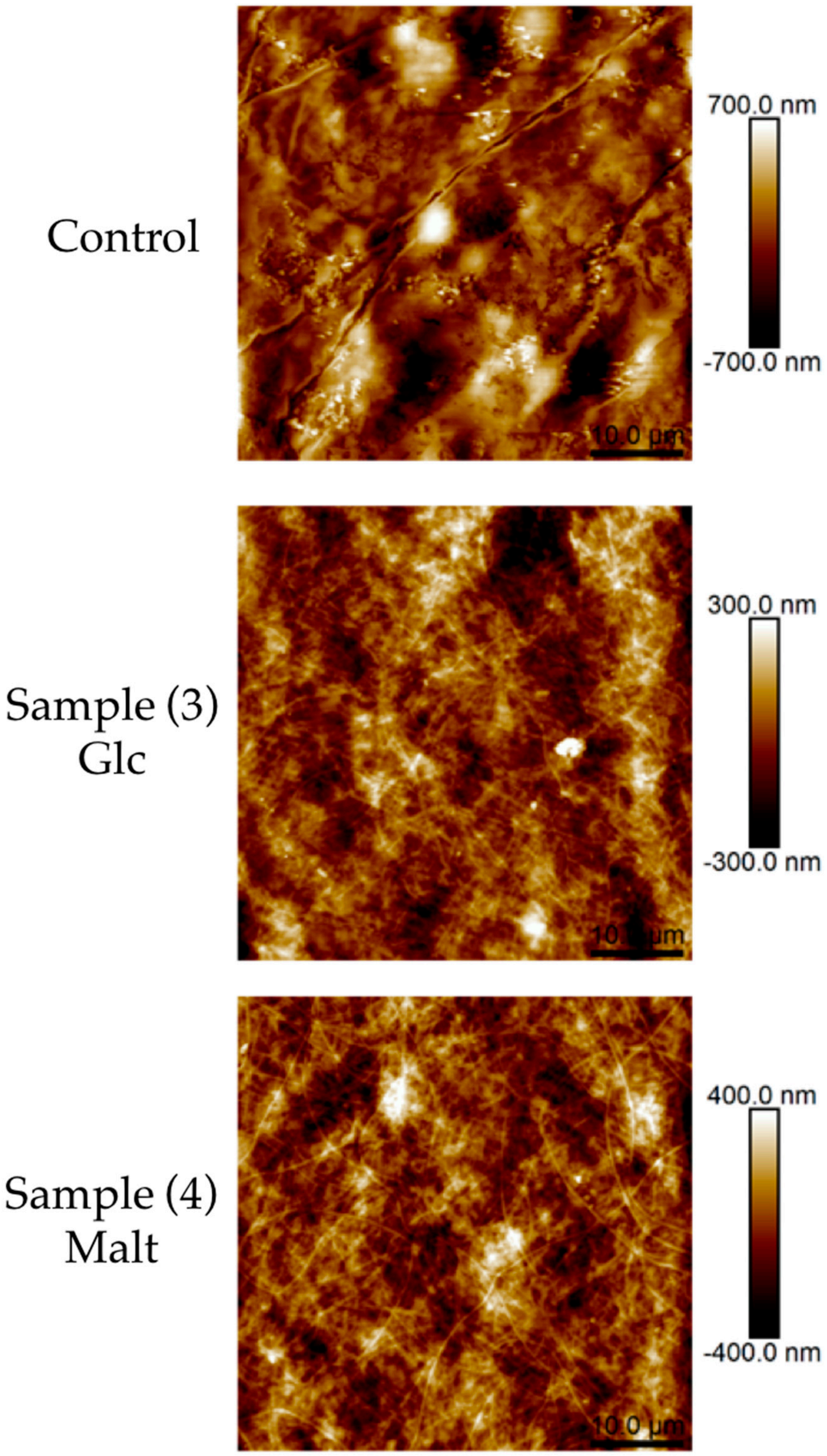

Figure Supplementary 4:

50x50 μm2 AFM topography image of collagen films reported in the main text (Figure 4) with variable z-range, from top to bottom: no-functionalized films, glucose and maltose neoglycosylated collagen films.

2. AFM Surface Roughness

The roughness of the samples was calculated in according with the standard formula $R_q = \sqrt{\frac{\sum Z_i^2}{N}}$ by using the available tool of the commercial Nanoscope Analysis software (Bruker Corporation, Santa Barbara, CA). The images were splitted into 25 different regions and the resulting surface roughness are evaluated and averaged (values reported in the main text are mean value ± st.dev).

Also the Ra for each sample was evaluated by the means of the standard formula $R_a = \frac{1}{N}\sum_{j=1}^{N} |Z_j|$ reporting values showing the same behavior than the ones found for the Rq (Ra = 165 ± 36 nm for CT, Ra = 59 ± 12 nm for Sample 3 and Ra = 88 ± 18 nm for Sample 4).
